# A cross-sectional study of submacular thickening in intermediate uveitis and determination of treatment threshold

**DOI:** 10.1186/s12886-016-0230-4

**Published:** 2016-05-18

**Authors:** Bruno Simonazzi, Konstantinos Balaskas, Yan Guex-Crosier

**Affiliations:** Jules-Gonin Eye Hospital, University of Lausanne, FAA, Av. de France 15, CH-1004 Lausanne, Switzerland; Manchester Royal Eye Hospital, Manchester, UK

**Keywords:** Optical coherence tomography (OCT), Intermediate uveitis, Pars planitis, Cystoid macular edema, Fluorescein angiography

## Abstract

**Background:**

The aim of this work is to refine understanding of anatomical and functional alterations in eyes with Intermediate Uveitis (IU), their natural history in mild cases not necessitating treatment and their response to treatment in severely affected eyes with macular edema.

**Methods:**

61 consecutive patients with IU presenting over a 6-year period were prospectively recruited into the study. Two subgroups of patients with IU were identified on the basis of the need or not for systemic cortico-steroid treatment. A group of healthy volunteers was identified for determining normal average central foveal thickness (CFT) values. Statistical comparisons were sought between patient sub-groups and with the group of normal volunteers for CFT and Best Corrected Visual Acuity (BCVA) at baseline and after 6 months. In a post hoc analysis, a cut-off value of CFT for systemic treatment initiation in IU was statistically identified and its sensitivity and specificity determined.

**Results:**

A statistically significant difference in mean CFT at baseline was observed between patients under systemic treatment and untreated patients (*p* = 0.0005) as well as between untreated patients and healthy volunteers. (*p* < 0.001) After six months difference in CFT between the two patients subgroups was no longer significant (*p* = 0.699). BCVA was worse for patients under systemic treatment. No statistically significant difference could be identified between the subgroup of untreated patients and the group of healthy volunteers either at baseline or after 6 months. Correlation between LogMAR visual acuity and central retinal thickness at baseline was strong (*r* = 0.7436, *p* < 0.0001, Pearson’s correlation coefficient). The cut-off value of CFT for initiating systemic treatment was determined at 215.5 μm in a post hoc analysis (sensitivity 62.5 %, specificity 96.4 %).

**Conclusions:**

Subclinical retinal thickening of mildly inflamed eyes with IU can occur though bearing no functional clinical significance and spontaneously resolving within 6 months. A cut-off CFT value for treatment of macular edema in IU, in the presence of other relevant morphological features on Optical Coherence Tomography, seems to emerge from post hoc analysis of collected data demonstrating strong specificity and moderate sensitivity.

## Background

Intermediate uveitis (IU) is a form of chronic ocular inflammation in which the vitreous is the ocular tissue predominantly affected, according to the SUN classification [1]. It usually involves a younger age range and comprises around 10 % of all causes of uveitis. Although unanswered questions persist concerning its etiology, the condition is occasionally associated with certain systemic pathologies, such as sarcoidosis, Lyme disease, Cat-scratch disease and multiple sclerosis [2]. The term pars planitis is used to describe the idiopathic form of IU characterized by the presence of snow-banking and/or snowballs in the vitreous [1]. Common manifestations of IU include retinal vasculitis, mainly involving venous branches and the development of cystoid macular edema (CME). The latter constitutes the principal factor leading to permanent visual loss in the context of IU and its frequency ranges between 30 and 60 % in several studies [2–4]. Despite the high incidence of CME, visual prognosis in IU is favorable with 75 % of patients retaining a visual acuity of 20/40 or better after ten years [3]. Poor visual prognosis is mainly associated with the development of CME, hence the importance of its early detection and treatment. A plethora of reports have underpinned the dominant role of Optical Coherence Tomography (OCT) for the diagnosis of macular edema in the context of ocular inflammation, including IU, and have identified quantitative and qualitative predictors for visual outcome [5–9] and response to treatment [10–12]. In the present prospective study we attempt to refine understanding of the natural history of CME in mild cases of IU not requiring systemic treatment as well as the response to treatment of more severe vision-threatening cases. We emphasize particularly the natural history and clinical significance of subclinical macular thickening not previously described in the context of this condition and we attempt to identify a threshold as regards CFT on OCT for initiating systemic cortico-steroid treatment.

## Methods

**Fig. 1:**
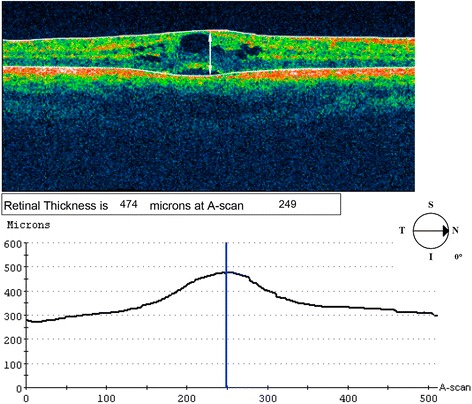
OCT Measurement of central foveal thickness. A cross sectional scan was performed at the macula (white double arrow). Central foveal thickness was determined by automated segmentation of retinal layers and positioning of the marker at the apex of the curve in the thickness chart. Central foveal thickness of 249 microns was measured in this case

We conducted a prospective study on patients presenting to the Jules - Gonin Eye Hospital with IU, diagnosed on the basis of clinical criteria proposed by the SUN classification [1]. Inclusion criteria also involved willingness of the patient to offer informed consent and age range between 9 and 77 years. Exclusion criteria were media opacities interfering with reliable OCT measurements and the presence of any other ocular condition associated with CME. All research work adhered to the tenets of the declaration of Helsinki. All patients underwent a full clinical examination, including best-corrected visual acuity (BCVA), tonometry and dilated fundus examination by indirect ophthalmoscopy with pars plana indentation. The following laboratory tests were performed on all patients: complete blood count, erythrocyte sedimentation rate, serum lysozyme, angiotensin converting enzyme, calcium and serologies for syphilis, Lyme disease, Cat-scratch disease. A quantiferon test or a tuberculin skin test to rule out tuberculosis and a chest X-ray were also performed. Fluorescein angiography was performed in all patients presenting with a visual acuity of 0.8 or worse or manifesting clinical signs of vasculitis, severe vitritis or macular edema. The decision to introduce a systemic treatment was based on clinical, OCT and/or angiographic criteria. More precisely, systemic cortico-steroid therapy was introduced in the presence of a loss of visual acuity of one or more lines (each line on the standardized visual acuity chart increasing by 0.1 log units), macular edema detected clinically or on OCT (including any presence of intraretinal cysts, irrespective or central retinal thickness), signs of moderate to severe vasculitis on clinical examination or angiography, more than 2+ of vitreous cells or more than 1+ of vitreous haze (assessed clinically on the basis of the SUN) classification, peripheral serous retinal detachment or papillitis. Systemic corticosteroids were used as first line therapy. Posterior subtenon’s steroid injections were used as second line therapy in view of the commonly bilateral nature of IU and the potential steroid responsiveness to subtenon’s injections. Steroid sparing agents were introduced in a second line therapy, when corticosteroids could not be tapered off after more than 3 months according to international guidelines. None of the patients were on immunosuppressive therapy during their inclusion in the study. A Stratus OCT was used (*Zeiss Humphrey Systems, San Leandro, CA*) with a cross-sectional scan performed at the macula to measure CFT (Fig. 1). In the presence of bilateral ocular inflammation, only the more severely inflamed eye was considered as the study eye, so as to account for inter-eye correlation in cases of bilateral involvement. The same evaluations as the ones performed at baseline were repeated 5 ± 1.6 months after initial evaluation. A group of 27 age-matched healthy volunteers without any ocular disease was included in the study. 6–10 OCT measurements per eye of the healthy volunteers were performed and averaged to determine normal values of central foveal thickness. For the purposes of refining understanding of disease behaviour, two sub-groups of patients were considered on the basis of the need or not of systemic treatment for IU. The first sub-group consisted of patients to whom systemic corticosteroids were administered and the second of patients without treatment, other than a short course of topical corticosteroids. This consisted of a course of prednisolone 1 % drops QDS on a tapering regimen over a period of 4–6 weeks. The amount of topical steroids did not vary between the two patient groups. Statistical significance of difference in mean CFT between the two patient sub-groups, as well as between each patient sub-group and the group of healthy volunteers was assessed at baseline and at the end of follow-up. The same statistical analysis was performed for difference in mean BCVA between the various groups at baseline and at the end of follow-up. The correlation between visual acuity and central foveal thickness at baseline was also evaluated. For purposes of statistical analysis Snellen visual acuity values were converted to the LogMAR scale. A post hoc analysis was performed in order to identify a cut-off value of CFT for initiating systemic cortico-steroid treatment. The sensitivity and specificity of this cut-off value were evaluated against the actual presence or absence of severe disease warranting systemic treatment in this patient cohort. Mann-Whitney-Wilcoxon test for independent samples (MWW), Wilcoxon matched pairs test and Pearson’s correlation coefficient were appropriately applied. Level of statistical significance was set to 5 %. Statistical analysis was performed with the use of the JMP software (JMP 8.0.1 copyright 2009 SAS institutes (www.jmp.com)).

## Results

The study sample consisted of 61 consecutive patients, of which 31 males and 30 females with a mean age of 30 ± 17 years, prospectively followed for a period of 6 months. There were 57 patients with pars planitis, 2 were diagnosed with sarcoidosis and 2 with Lyme disease. 29 patients did not receive systemic treatment, while 32 patients received systemic cortico-steroid treatment with an initial dosage of 1 mg/kg/day. A progressive tapering regimen was used with a decrease of 5 mg every three to four weeks. The control group of 27 healthy subjects consisted of 12 men and 15 women with a mean age of 36 ± 16 years ((*p* = 0.139) for mean age difference between patient and control group, *t*-test). All patients were phakic. CFT was 176 ± 16 μm for the normal volunteers (*n* = 27), while it was 310 ± 182 μm for the group of patients under systemic treatment (*n* = 32) and 183 ± 24 μm for the group of untreated patients (*n* = 29). Difference in CFT at baseline was statistically significant between normal subjects and untreated patients (*p* < 0.0001, MWW), normal subjects and patients under systemic treatment (*p* < 0.0001, MWW) and between treated and untreated patients (*p* = 0.0005, MWW) [Table 1].

**Table 1:** Statistical significance of difference in central retinal thickness between the various groups

	Untreated patients	Treated patients		Control group
Untreated patients		Baseline *p* = 0.0005	6 months *p* = 0.699	Baseline *p* < 0.0001
Treated patients				Baseline *p* < 0.0001
Control group				

*p*-values derived from Mann-Whitney-Wilcoxon test

15 patients did not complete the full 6 months follow-up period (8 patients in the group of untreated patients and 7 patients in the group of systemically treated patients). For patients available for the 6-months follow-up visit (mean CFT of 325 ± 122 at baseline) mean CFT decreased in a statistically significant manner to 209 ± 86 μm for the group of treated patients (*n* = 25, *p* = 0.0004, Wilcoxon matched pairs), while it remained practically unchanged in the group of untreated patients (181 ± 29 μm from 179 ± 18 at baseline) (*n* = 21, *p* = 0.561, Wilcoxon matched pairs). Difference in CFT was no longer statistically significant between the two patient subgroups (*p* = 0.699, MMW).

Mean initial BCVA differed in a statistically significant manner between the two patient sub-groups (*p* < 0.0001, MWW), but not between untreated patients and the group of normal volunteers (*p* = 0.085, MWW) [Table 2]. Mean BCVA slightly, though statistically significantly, improved in the group of treated patients, passing from 0.222 ± 0.249 to 0.087 ± 0.125 (*p* = 0.0075, Wilcoxon matched pairs), while it remained practically unchanged in the group of untreated patients, passing from 0.024 ± 0.085 to 0.015 ± 0.060 (*p* = 0.644, Wilcoxon matched pairs). Changes in CFT and BCVA for the two patient subgroups over the follow-up period are summarized in Table 3. Difference in BCVA between the two patient sub-groups remained statistically significant at the end of follow-up (*p* = 0.032, MWW). There was a strong correlation between baseline BCVA and CFT on OCT (*r* = 0.743, *p* < 0.0001 Pearson’s correlation coefficient). Comparisons bear on patients that completed the 6 months of follow up only. In the post hoc analysis a CFT cut-off for initiating treatment with systemic cortico-steroids was determined at 215.5 μm in the present cohort. The sensitivity of this value was moderate at 62.5 %, yet its specificity was strong at 96.4 %.

**Table 2:** Statistical significance of difference in LogMAR BCVA between the various groups

	Untreated patients	Treated patients		Control group
Untreated patients		Baseline *p* < 0.0001	6 months *p* = 0.032	Baseline *p* = 0.085
Treated patients				Baseline *p* < 0.0001
Control group				

*p*-values derived from Mann-Whitney-Wilcoxon test

**Table 3:** Statistical significance of change in central retinal thickness and BCVA between baseline/end of follow-up. (Patients who completed 6 months of follow-up)

	Baseline	6-months	*p*
Untreated patients			
Central foveal thickness (μm)	179 ± 18	181 ± 29	0.561
BCVA (LogMAR)	0.024 ± 0.085	0.015 ± 0.060	0.644
Treated patients			
Central foveal thickness (μm)	325 ± 122	209 ± 86	0.0004
BCVA (LogMAR)	0.222 ± 0.249	0.087 ± 0.125	0.0075

*p*-values derived from Wilcoxon matched pairs test

## Discussion

The present study accrues useful additional clues in our understanding of the anatomical and functional repercussions of IU and its response to cortico-steroid treatment. Segregating patients with respect to the need for systemic treatment for CME and comparing anatomical and functional outcomes between patient sub-groups and with a group of healthy volunteers in a prospective study design allowed identifying the presence of subclinical retinal thickening in milder, not systemically treated cases as well as the clinical significance of this phenomenon. Re-affirming the established favorable prognosis to systemic treatment of CME secondary to IU, there was a statistically significant decrease in CFT after a period of 6 months in the sub-group of more severely affected patients, which translated into a significant, though slight, improvement in BCVA. Although in the group of patients that did not receive systemic treatment mean CFT was significantly inferior to that of the treated group, it was, nevertheless, significantly superior to that of the group of healthy volunteers. Despite this marginal retinal thickening in the group of untreated patients, retinal thickness did not increase further in the course of follow-up, nor was there a significant drop in visual acuity suggesting no clinical significance of subclinical retinal thickening in milder cases of IU. This slight, though statistically significant, macular thickening in the group of patients with mild disease, not necessitating systemic treatment, is a striking finding not previously identified in the context of IU. This patient sub-group presented sub-clinical macular thickening, as evidenced by the absence of visual acuity loss in these patients, compared to the group of normal volunteers. Few studies have tackled the issue of subclinical retinal thickening in other forms of intraocular inflammation, such as acute anterior uveitis (AAU) [13, 14]. These include a retrospective study by Castellano [13], demonstrating a statistically significant difference in retinal thickness between the study and fellow eye for all OCT subfields in patients with acute anterior uveitis and the prospective study by Balaskas et al. [15] that offered a model of quantification of retinal thickness asymmetry between fellow eyes in the course of an episode of AAU. The identification of a critical value of retinal thickness beyond which systemic treatment is clearly indicated in patients with IU has yet to be determined, though it would constitute a powerful clinical tool. Although a precise CFT threshold for treatment cannot be envisaged in isolation, our post hoc analysis determined a cut-off value of 215.5 μm for commencing treatment in the present cohort. Clinical management decisions cannot be based on CFT values alone, however CFT should be taken into consideration when reaching management decisions alongside other qualitative morphological features on OCT, most importantly the presence of cystoid spaces. In the presence of cystic changes on OCT, however, determining a cut-off CFT value beyond which the risk-benefit ratio is tipped in favour of systemic treatment is of particular clinical relevance. When analysed against the actual presence or absence of severe disease requiring systemic treatment in our series, this cut-off value for CFT exhibited strong specificity, yet moderate sensitivity. This may indicate that cases requiring systemic treatment may occasionally be missed on the basis of this cut-off value if considered in isolation, while, on the other hand, this value is an accurate indicator of severe disease warranting systemic treatment in the vast majority of such cases. Taking into account that in a real-life scenario, clinical management decisions will be based on a multitude of factors rather than on CFT alone, the strong specificity of this cut-off value may offer a useful tool to clinicians as an additional argument in favour of systemic treatment in relevant cases that are more likely to benefit from it. Strong correlation between CFT thickness and mean logMAR visual acuity at baseline was observed in the present study. The issue of correlation between retinal thickness and visual acuity remains controversial with some studies reporting a high [16] negative correlation and others failing to verify this finding [17]. Several factors may play a role in the observed discrepancy, such as structural characteristics of macular edema, chronicity and underlying pathology. The high specificity of the identified CFT cut-off value for initiating systemic treatment in this study suggests that almost all cases deemed of sufficient severity to warrant systemic treatment in our practice, on the basis of various clinical indicators and not just OCT findings, had a CFT that exceeded the determined cut-off value. Although clinical application of this cut-off CFT value needs to be in conjunction with other clinical and imaging features rather than in isolation, its high specificity renders it a useful adjunct in the decision making process for commencing systemic treatment in IU. From a practical point of view, CFT values can be obtained on newer generation OCT technology as well and can be evaluated against the cut-off value suggested in this work employing conversion algorithms already reported in the literature, hence retaining their clinical relevance and usefulness [18, 19]. The present study has certain limitations. Patients with advanced media opacities preventing clear visualization of the fundus and interfering with OCT measurements were excluded. The use of CFT as a marker of retinal thickness, although not uncommon in the literature, is a rather less frequently used clinical endpoint for research when compared to the more familiar central macular thickness (CMT) value. The choice of CFT in this work as clinical endpoint resides in the researcher’s reliance on this marker for clinical decision making in the era of the Stratus OCT. The age-matched group of healthy volunteers randomly selected may not be representative of the general population for determining normal CFT range, although very similar values for CFT in healthy eyes have been previously reported [20].

## Conclusions

Present study contributes two novel concepts in our understanding of macular thickening in the context of IU:

1. Subclinical retinal thickening of mildly inflamed eyes with IU is a previously unreported phenomenon, with however no functional clinical significance and spontaneous resolution by 6 months.
2. A cut-off value of CFT for initiating systemic treatment of 215.5 μm was identified in the present cohort exhibiting strong specificity and moderate sensitivity. This may serve as a useful additional clinical tool aiding clinicians in reaching therapeutic decisions in patients with IU.

## Ethics approval and consent to participate

Ethics approval was obtained by the Ethics Committee of the University of Lausanne, Switzerland (reference number VD 33/09). Informed consent has been obtained for all participants. For patients under 16 years of age, consent has been obtained from their parents/guardians.

## Consent for publication

Not applicable (no identifying patient data).

## Availability of data and materials

Data will be shared upon request.

## Abbreviations

AAU: acute anterior uveitis
BCVA: best-corrected visual acuity
CME: cystoid macula edema
CFT: central foveal thickness, mean thickness at the intersection of radial scans
CMT: central macular thickness
IU: intermediate uveitis
LogMAR: log (decimal acuity) = −log of the minimal angle resolution
MWW: Mann-Whitney-Wilcoxon test
OCT: optical coherence tomography
SUN: standardization of uveitis nomenclature (SUN)
